# Use of nicotine vaping products during an attempt to quit smoking by Canadian adults who smoke or recently quit: findings from the 2022 Canada International Tobacco Control Four Country Smoking and Vaping Survey

**DOI:** 10.24095/hpcdp.45.1.04

**Published:** 2025-01

**Authors:** Shannon Gravely, David Sweanor, Pete Driezen, David T. Levy, Geoffrey T. Fong, Anne C. K. Quah, Lorraine V. Craig, Janet Chung-Hall, Susan C. Kaai, K. Michael Cummings

**Affiliations:** 1 Department of Psychology, University of Waterloo, ON, Canada; 2 Faculty of Law, University of Ottawa, ON, Canada; 3 Centre for Health Law, Policy and Ethics, University of Ottawa, ON, Canada; 4 School of Public Health Sciences, University of Waterloo, ON, Canada; 5 School of Medicine, Georgetown University, Washington, DC, USA; 6 Ontario Institute for Cancer Research, Toronto, ON, Canada; 7 Department of Psychiatry and Behavioral Sciences, Medical University of South Carolina, Charleston, SC, USA

**Keywords:** nicotine vaping, cigarette smoking, quit attempt, vaping flavours, vaping devices, policy

## Abstract

An analysis of 1771 Canadian adults who smoke or used to smoke cigarettes was conducted using data from the 2022 International Tobacco Control Four Country Smoking and Vaping Survey. Using weighted data, we estimated the prevalence of Canadian adults who tried to quit smoking between 2020 and 2022, and the use of a nicotine vaping product (NVP) and the flavours and devices used most often at their most recent quit attempt. Overall, 36.5% made a quit attempt; of those, 19.4% used an NVP. Those who were younger and quit smoking were more likely to have used an NVP. Prefilled cartridges or pods (36.3%) and fruit flavours (39.5%) were used most frequently.

**Highlights**

- In 2021, Health Canada proposed imposing federal restrictions on all nicotine vaping product (NVP) flavours except for tobacco, menthol and mint, although some provinces have already implemented flavour bans.
- One in five Canadian adults who tried to quit smoking used an NVP during their most recent attempt; fruit flavours and prefilled cartridges or pods were most commonly used.
- 68% of Canadian adults who attempted to quit smoking used flavours that would be prohibited under Health Canada’s flavour restrictions proposal.
- There were no differences in flavours or devices used most between those who reported quitting smoking and those who did not quit.

## Introduction

Cigarette smoking causes about 48000 deaths in Canada each year,^1^ and 3.8 million Canadians smoked cigarettes in 2022.^2^ Canadian clinical practice guidelines state that the most effective smoking cessation method is a combination of pharmacotherapy and face-to-face behavioural support from a health care professional.^3^ However, few people use prescription medication and support services.^4^,^5^ Although nicotine vaping products (NVPs, also known as e-cigarettes) are not approved as a smoking cessation aid in Canada, they have been found to be effective in helping people to quit smoking,^6^ particularly when vaping is more frequent (e.g. daily).^7^-^9^ The Canadian government has stated that “switching completely to vaping means stopping smoking all cigarettes, which will reduce the risks of harms to your health.”^10^

The increase in vaping among youth and non-smoking young adults in Canada^11^ is a significant public health concern.^12^-^16^ The availability of a variety of flavours, coupled with novel, innovative and inexpensive devices likely appeals to tech-savvy youth and young adults. As a result, some provinces have adopted or are considering regulations intended to deter NVP use by minors.^15^,^16^ In June 2021, Health Canada published a draft regulatory proposal with the intention of protecting youth from inducements to use vaping products, in order to help reduce youth vaping by (1)restricting the promotion of flavours in vaping products to tobacco, mint, menthol and a combination of mint and menthol; (2) prohibiting all sugars and sweeteners as well as most flavouring ingredients, with limited exceptions, to impart tobacco, mint, menthol, or a combination of mint and menthol flavours; and (3) prescribe sensory attributes standards to prevent a sensory perception other than one that is typical of tobacco or mint/menthol.^17^

Little is known about Canadian adults who used an NVP when they were most recently attempting to quit smoking except that they appear to prefer fruity and other sweet NVP flavours.^18^-^21^ Studies also suggest that adults who vape are less likely than youth to use disposable devices.^22^-^24^

Using data from a nationally representative survey of Canadian adults who smoke cigarettes or quit smoking, we estimated (1) the prevalence of adults who attempted to quit smoking between 2020 and 2022; (2) the use of NVPs by those who attempted to quit smoking; and (3) the NVP flavour and device used most when they were attempting to quit.

## Methods

The current study used data from Wave 4 (August–December 2022) of the Canadian arm of the International Tobacco Control (ITC) Four Country Smoking and Vaping (4CV) Survey and included Canadian adults (≥18 years) who reported that they smoked cigarettes daily (n = 1217), weekly (n = 262) or monthly, but had previously smoked daily (n = 65) or quit smoking in the last 2 years (and had previously smoked daily or smoked at least weekly in the last 24months; n = 227).

Respondents were recruited from Leger Opinion’s (Montral, QC) online probability-based panel across 10 provinces. All eligible respondents provided consent.

***Ethics approval***

The survey protocols and all materials, including the survey questionnaires, were approved by the University of Waterloo Research Ethics Board (REB#20803/30570). Details about the 2022 *ITC 4CV Survey are presented in the ITC Four Country Smoking and Vaping Survey, Wave 4 (4CV4, 2022) Technical Report*.^25^

***Measures***

The 2022 Canadian ITC 4CV Survey questionnaire is available from the ITC Project website: https://itcproject.org/surveys/canada/4cv4-ca/.

Respondents were asked, “In the last 24months, have you tried to stop smoking?” If they answered “yes,” they were then instructed to “select all that apply: Which of the following forms of help did you use as part of your last quit attempt?” The response options considered for this study were a vaping product (e-cigarette); nicotine replacement therapy (NRT); prescription medications (combined: varenicline or bupropion); and/or support services (combined: telephone quitline service or smokers’ helpline, apps or automated services on a mobile phone or tablet, and/or clinic, individual or group counselling, stop-smoking course or behaviour therapy); or quitting on their own without using any medication, nicotine (such as e-cigarettes, heated tobacco or smokeless tobacco products, nicotine pouches), support services or other methods of assistance (i.e. no assistance). Respondents could select more than one form of assistance, if applicable. If respondents reported using a vaping product (e-cigarette) at the time of their most recent quit attempt, they were asked: (1) “What type of vaping device did you use on your last quit attempt”; and (2) “Which e-liquid flavour category did you use most for your last quit attempt”?

***Statistical analyses***

Weighted descriptive statistics were used to estimate the proportion of Canadian adults who attempted to quit smoking between 2020 and 2022. Cross-sectional weights were computed to make the sample as representative as possible of the Canadian adult population who vape, smoke or formerly smoked, with respect to sex, age group, education and geographic region. The 2022 Canadian Tobacco and Nicotine Survey was used as the benchmark for the construction of the weights.

We identified the population characteristics of those who were more likely to use an NVP at their most recent quit attempt. We used multinomial regression to assess the flavour and device used most often by those who used an NVP at the time of their quit attempt, adjusting for age, sex and smoking status. Thereafter, we used a logistic regression model to compare whether there were differences by smoking status in the use of flavours that would be prohibited versus those that would not under the Health Canada flavour restrictions proposal. The model adjusted for sex and age.

***Data availability statement***

In each country participating in the ITC Policy Evaluation Project, the data are jointly owned by the lead researcher(s) in that country and the ITC Project at the University of Waterloo. Data from the ITC Project are available to approved researchers 2 years after the date of issuance of cleaned data sets by the ITC Data Management Centre. Researchers interested in using ITC data are required to apply for approval by submitting an International Tobacco Control Data Repository (ITCDR) request application and subsequently to sign an ITCDR Data Usage Agreement. The criteria for data usage approval and the contents of the Data Usage Agreement are described online (http://www.itcproject.org).

## Results

Of the 1771 adults who were eligible for inclusion for further analyses, 36.5% (weighted; n = 739) reported that they attempted to quit smoking at least once in the last 2 years; 37.4% did not use any assistance, 31.2% used NRT, 19.4% used an NVP, 12.2% used prescription medication (varenicline or bupropion) and 8.8% used support services.

Those who used an NVP when they most recently tried to quit were more likely to be younger (18–39 years; *p* <0.001) and to report having quit smoking (31.5%; *p*<0.001) and used support services (34.9%; *p* = 0.03) (see Table 1). Of those respondents who used an NVP (n = 169), 45.5% also used NRT (n = 61), 13.5% used a prescription medication (n = 22) and 20.7% used support services (n = 21).

**Table 1:** Characteristics of adults (≥18 years) who used an NVP versus those who did not use an NVP during their most recent smoking quit attempt between 2020 and 2022,a Canada (n = 739)

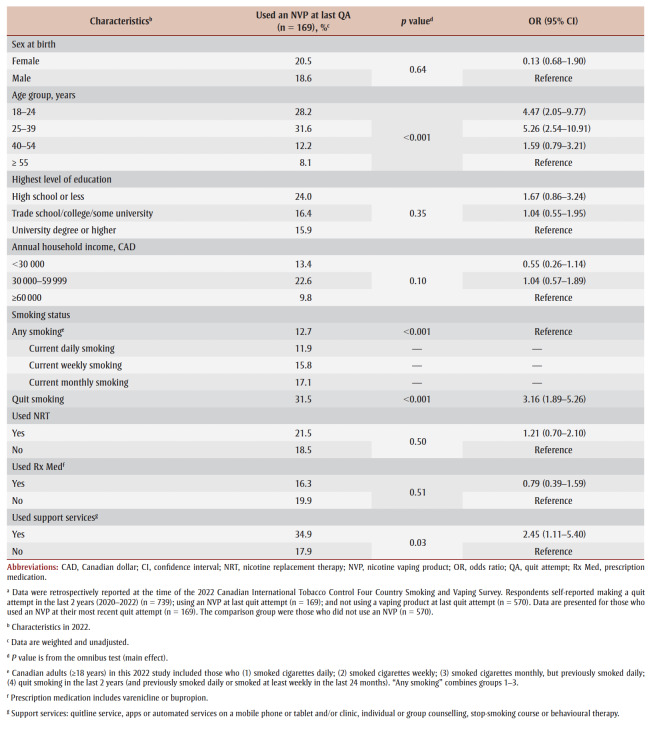

The most commonly used device types were prefilled cartridge or pod devices (36.3%) and the most commonly used flavours were fruit flavours (39.5%) (see Table 2). There were no significant differences between those who smoked or quit smoking by device type (p = 0.74) or flavour (p = 0.36). The regression analysis found that a majority of adults (both those with a failed and successful quit attempt) used flavours that would be prohibited (67.6%) under the proposed Health Canada flavour restrictions.

**Table 2:** Device types and flavours used most by adults (≥18 years) who used an NVP during their most recent smoking quit attempt between 2020 and 2022, Canada (n = 169)

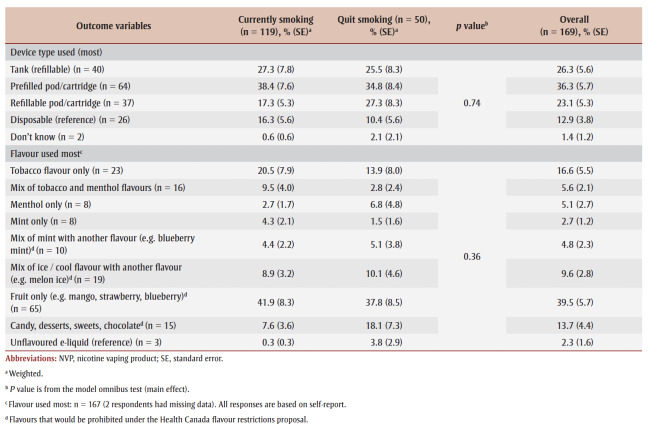

A higher percentage of those who quit smoking used e-liquid flavours that would be prohibited (70.9%) than those who were smoking in 2022 (66.3%), but the difference was not statistically significant (*p* = 0.70) (see Table 3).

**Table 3:** Comparison of current and former smokers (≥18 years) who used an NVP with flavours that would be prohibited versus those that would not be prohibiteda during their most recent smoking quit attempt between 2020 and 2022, Canada (n = 169)

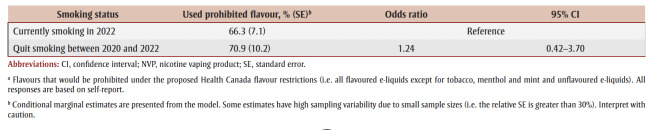

## Discussion

We found that about two in five Canadian adults who smoked cigarettes tried to quit between 2020 and 2022. Nearly 40% of those who tried to quit did not use any form of assistance. Of those who tried to quit with assistance, NRT was the most common form of assistance reported, followed by NVPs. Close to half of the adults who used an NVP also used NRT when they most recently tried to quit. Those who were more likely to use an NVP were younger and reported having quit smoking. There were no significant differences between adults who failed and succeeded quitting when using an NVP in terms of device types or flavours. However, the majority of all adults used flavours that would be prohibited under Health Canada’s flavour restrictions proposal. This raises the possibility of unintended consequences of policies that would make NVPs less appealing and satisfying as substitutes for cigarettes, which might diminish initiation and maintenance of NVP use by adults who smoke and are considering switching to an NVP.

Because of the dangers of cigarette smoking, health care providers should encourage individuals who smoke to use whatever method is necessary to stop smoking. For those attempting to quit without assistance (i.e. “cold turkey”), the failure rate for a given quit attempt is typically greater than 90%.^5^ Notably, while NVPs may offer an effective way for people who smoke to transition away from cigarettes,^6^-^9^,^26^ even among those who do not initially plan to quit,^27^ NVP preferences when trying to quit smoking vary.^4^ For example, some adults (particularly those who are older) may not consider using an NVP as a cessation aid, but may prefer to use other forms of assistance. Using pharmacological treatment in any capacity can significantly increase the chances that tobacco-dependent adults will successfully quit smoking.^5^ Complete substitution with NVPs may also help individuals remain abstinent, but this requires more investigation.

Evidence suggests that e-liquid flavours are an important factor in the initiation, maintenance, acceptability, appeal and satisfaction related to e-cigarette use.^19^,^28^-^30^ The “taste” of flavours is a subjective sensory experience that varies from individual to individual as a result of different threshold sensitivities.^31^ While some people prefer harsh, bitter or sour flavours, others favour sweet, savoury or cool flavours. The aim of our study was not to test differences in NVP flavours used on smoking cessation outcomes, but rather to describe preferences of NVP flavours used most by Canadian adults who have attempted to quit smoking. Our study found that adults specifically seeking to quit smoking showed a strong preference for flavoured products that would be restricted under the current Health Canada policy proposal. A smaller, but nonnegligible, proportion of adults most often used tobacco flavour, although this was more common among older adults.

Making lower-risk products less appealing should be carefully considered in policy development, including any impact in diminishing the interest in, and successful use of, NVPs by Canadian adults when trying to achieve smoking cessation. Given the likely importance of flavoured NVPs for adults, and to also prevent youth use or uptake, alternatives to an outright flavour ban should be considered, such as requiring that NVPs be sold by licensed adult retailers under regulations requiring plain packaging, strict regulations on advertising and promotions, and strict age verification at the time of purchase. To encourage transitions from combusted to non-combusted products, risk-proportionate regulation and taxation should be deployed.

***Strengths and limitations***

This study has some limitations. First, being cross-sectional, this study cannot be used to infer causality (e.g. we cannot determine whether certain flavours or devices were causally related to cessation outcomes). Second, the retrospective measurements in this study may have resulted in recall bias. Finally, some estimates should be interpreted with caution due to small sample sizes in some subgroups.

## Conclusion

Overall, we found that most of the adults who attempted to quit smoking and used an NVP were using a variety of flavours that would be restricted under the Health Canada vaping flavour ban policy. Careful consideration should be given to the effects of policies that would ban appealing flavoured NVP products from the market. Prospective research studies are needed to examine the role of flavours specially for smoking cessation purposes.

## Funding

The ITC Four Country Smoking and Vaping Surveys were supported by grants from the US National Cancer Institute (P01 CA200512) and the Canadian Institutes of Health Research (FDN-148477). GTF receives a Senior Investigator Grant from the Ontario Institute for Cancer Research. The funders had no role in the design of the study, in the collection, analyses or interpretation of data, in the writing of the manuscript, or in the decision to publish the results.

## Conflicts of interest

KMC has served and continues to serve as a paid expert witness in litigation against cigarette manufacturers. GTF has served as an expert witness or consultant for governments defending their country’s tobacco policies or regulations in litigation and was a member of the Health Canada Vaping Products Scientific Advisory Group (2017–2020; unpaid). All other authors have no conflicts of interest to declare.

## Authors’ contributions and statement

SG: Conceptualization, formal analysis, writing – original draft.

DS: Conceptualization, writing – review & editing.

PD: Data validation, writing – review & editing.

DTL: Writing – review & editing.

GTF: Funding acquisition, writing – review & editing.

ACKQ: Writing – review & editing.

LVC: Writing – review & editing.

JC: Writing – review & editing.

SCK: Writing – review & editing.

KMC: Funding acquisition, writing – review & editing.

All authors approved the final manuscript prior to submission.

The content and views expressed in this article are those of the authors and do not necessarily reflect those of the Government of Canada.
